# The Role of Extrinsic Rewards and Cue-Intention Association in Prospective Memory in Young Children

**DOI:** 10.1371/journal.pone.0140987

**Published:** 2015-10-21

**Authors:** Daniel Patrick Sheppard, Anett Kretschmer, Elisa Knispel, Bianka Vollert, Mareike Altgassen

**Affiliations:** 1 Donders Institute for Brain, Cognition and Behaviour, Radboud University, Nijmegen, The Netherlands; 2 Department of Psychology, Technische Universitaet Dresden, Dresden, Germany; University College London, UNITED KINGDOM

## Abstract

The current study examined, for the first time, the effect of cue-intention association, as well as the effects of promised extrinsic rewards, on prospective memory in young children, aged 5-years-old (n = 39) and 7-years-old (n = 40). Children were asked to name pictures for a toy mole, whilst also having to remember to respond differently to certain target pictures (prospective memory task). The level to which the target picture was associated with the intention was manipulated across two conditions (low- or high-association) for all participants, whilst half of the participants were promised a reward for good prospective memory performance. Results showed a main effect of age, with the 7-year-olds outperforming the 5-year-olds. Furthermore, there was a main effect of reward, with those promised a reward performing better than those who were not. No effect was found for cue-association, with the participants of both age groups performing equally well in both association conditions. No significant interactions were found between any of the variables. The potentially important role of reward in young children’s everyday prospective memory tasks, and possible reasons for the lack of a reflexive-associative effect, are discussed.

## Introduction

Prospective memory (PM) tasks are tasks in which planned intentions must be executed in the future [1] either upon the occurrence of an event (event-based PM) or at a certain time (time-based PM). These tasks are critical to everyday life, with failure possibly resulting in serious personal and social consequences (e.g. missing medication or forgetting to pass on an important message to a supervisor) and for children this is no different (e.g. forgetting to bring homework and/or equipment to school, missing a school trip). Indeed, for young children starting school, there is arguably a sudden increase in PM demands, and an expectation by adults to behave increasingly independently [2]. As it has been shown that children who develop poor PM abilities are likely to experience difficulties in interacting with parents, teachers, and peers [3, 4] it is clear that those starting school are at a critical stage in their development, and it is thus important to investigate PM in this age group. The purpose of the current study, therefore, was to further elucidate upon the development of PM in young children and the possible underlying cognitive mechanisms. This could lead to a better understanding of the conditions under which successful PM can be achieved, even at a young age.

That PM ability develops and improves with age is now well-established [5–11]. However, the mechanisms driving this development, and the conditions under which the age effects are most pronounced, are still unclear. In their recent review, Mahy, Moses [12] put forward their executive model of PM development, positing that executive functions (EF) are the most important mechanisms underlying PM development. EF comprises abilities such as set-shifting, inhibition and working memory. The authors argue that the well-established and protracted development of EF abilities, which improve from early childhood [13–15] through to adulthood (see [16] for a review), are instrumental in the developing ability to execute an intention at the appropriate time/event in the face of a distracting ongoing task (OT). This view is complimented by the influential Multiprocess Framework [17] which states that PM tasks vary in the type of retrieval processes required and can rely either on rather automatic (low EF demand) or strategic (high EF demand) processes. For instance, some tasks will automatically ‘pop into mind’ on presentation of a PM cue (e.g., if the cue is distinctive/salient, or if it is strongly associated with the intention, such as a red letter box prompting memory of the intention to post a letter) whilst others will necessitate a strategic monitoring of the environment for the PM cue (e.g., if the cue is not presented in the immediate environment, such as looking out for a pharmacy whilst driving home). Furthermore, those tasks which demand a higher degree of strategic monitoring will leave less available resources for the OT, resulting in costs to OT performance [18]. This Multiprocess Framework is supported by evidence from studies with young children employing dual-task paradigms (i.e. participants completing an OT with an embedded PM task) which attempt to directly manipulate the need for automatic or strategic processes, and better understand the role of executive functioning at this age [5, 7–9, 19, 20]. For instance, in a series of computer-based experiments, Kliegel, Mahy [9] found that, when the need for strategic and executive processes was reduced by increasing the PM cue saliency (or distinctiveness), cue centrality (i.e. cue inside, rather than outside the centre of attention) and reducing OT absorption, PM performance was improved in 6- to 10-year-olds. Furthermore, they found that when cue centrality was increased, the younger children performed as well as the older children, a result which implies the age difference in performance was due to the additional strategic requirements of monitoring when the cue was outside the focus of attention. Mahy, Moses [20] also found that 5-year-olds outperformed 4-year-olds, and that performance for both age groups was worse when cues were less salient and the OT more difficult. Further, the authors reported that the EF ability, inhibition, measured via performance on the Simon Says task [13] accounted for a significant level of variance for non-salient cues, and individual differences in inhibition fully mediated the effect of age on PM performance. In another study, Kvavilashvili, Messer [7] found that increasing inhibition demands adversely affected PM task performance. Specifically, the authors found that requiring the children to interrupt the OT, in order to execute the PM task, resulted in worse performance than when the PM task was at the end of the OT. This result was further supported by similar findings from Kliegel, Mackinlay [19] who found the effects of interrupting the task to be greater for children of an average age of 7- and 10-years, and for older adults of around 67-years-old, than for younger adults 25-years of age, indicative of less developed EF in children, and reduced EF in older adults. Further supporting evidence for the role of EF for PM performance stems from studies using a correlational approach [8, 21, 22]. For instance, Mahy and Moses [8] showed that working memory significantly predicted PM (even after controlling for age and inhibition) in 4 to 6-year-old children.

In sum, according to the Multiprocess Framework [17] and Mahy et al.’s Executive Framework [12] and supporting evidence, younger children, whose EF resources are less developed, will find PM tasks that are high in EF demand more difficult. However, they can perform well on tasks which depend more on automatic, reflexive processes and hence are lower in EF demand or if the task encourages the allocation of their limited EF resources. It is important, then, to further investigate the role of EF in PM in children to better understand the conditions under which automatic processes, or resource allocation, can be encouraged and thus better support children in everyday life.

Two factors that have been put forward by the Multiprocess Framework to impact on PM performance are motivation and cue-intention association. To date only one study has investigated the effect of extrinsic *incentives* in PM in young children [23] and none have examined *cue-intention association* [24] in a younger age group, two factors which will be discussed, respectively, henceforth.

The benefit of motivation in PM in young children, particularly with regard to the use of incentives, is an area that has received very little attention in the literature. However, despite young children’s limited EF resources [25–28] some studies suggest that successful PM performance is possible for very young children if they are highly motivated [29–32]. Ślusarczyk and Niedźwieńska [30] for example, found that tasks which they considered to contain a high level of intrinsic motivation, such as reminding the experimenter to give them a candy at the end of session, as opposed to remembering to put pencils on the shelf, resulted in higher PM performance for all participating young children, ranging in age from 2- to 6-years-old. Causey and Bjorklund [32] also found that 2- to 4-year-olds were more successful in collecting a sticker at the end of the session, than they were at turning over a sign. Arguably, these results are in line with the Multiprocess Framework [17] and the Executive Framework [12] as highly motivating tasks demand less in terms of inhibition, and encourage the allocation of limited resources to the PM task. This notion is further supported by the goal-based motivational model posited by Penningroth and Scott [33] which suggests that PM performance will be improved when the intention has high personal relevance. It seems plausible then, that if motivation was achieved via more extrinsic motivators, such as incentives, then the same positive motivation effects could be expected. Indeed, positive effects of monetary incentives have recently been found in healthy adults [34] and adolescents with traumatic brain injury [35, 36]. However, only one study to date has investigated the effect of incentives, rather than the use of more intrinsically motivating tasks, on PM performance in young children [23] and they did not find a positive effect.

However, in Guajardo and Best’s [23] computer-based task, 3- and 5-year-olds were shown a series of 6 blocks of 10 pictures, with each picture shown individually for 5s, and a 1s between-picture interval, and were told they would be asked to recall as many pictures as possible after each block (OT). For the additional PM task, they were told to press the space bar every time they saw a target picture (one in each block). It could be argued that this is a difficult task for such young children as it loads heavily on working memory and demands prolonged attention, which could have had a negative impact on PM performance and the effect of the reward manipulation. Furthermore, children were provided with common food and toy items (e.g., pennies, fruit chews) *each time* they correctly pressed a key on presentation of a PM target picture, which may not have been very motivating. Therefore, it is the intention of the present study to reduce the executive demands of the OT and to provide a more motivating incentive.

A further factor yet to be studied in young children is that of the strength of cue-intention association. It is, however, a manipulation that McDaniel, Guynn [24] found to be effective in healthy adults, informing their ‘reflexive-associative hypothesis’, with the pertinent result being that PM was better when PM cues were highly associated with the intention (e.g. writing the word *thread* on presentation of the word *needle*) than when there was a low cue-intention association (e.g. *spaghetti-thread*). The authors argue that this finding is in line with the Multiprocess Framework [17], in that the low-association condition required strategic monitoring processes, whilst performance in the high cue-intention association condition reflected automatic, reflexive-associative retrieval. The positive effect of high cue-intention association has also been found in other studies, for example, in older adults [24, 37, 38] and clinical populations [39]. Woods, Dawson [39] for instance, found that the PM performance of HIV patients, a clinical group found to be particularly susceptible to age-related executive functioning and attentional impairment [40, 41], was better when cues and intentions were semantically related (e.g., “When I hand you a postcard, self-address it”) then when they were unrelated (e.g., “When I show you a picture of a cow, snap your fingers“). Following this line of reasoning, it could be expected that cue-intention association might play an important role in children’s PM performance; however, no study to date has examined this aspect in children, and is thus of interest to the current study.

The primary aim of the current study, therefore, was to investigate the role of cue-intention association and motivation in the PM ability of 5- and 7-year-old children, hence examining PM in age-groups at, or around, the age of starting school, who are thus experiencing increasing everyday PM demands. Children performed a simple card-naming OT, with the embedded PM task including both a high- and low-association condition. Further, half of the children were provided the incentive of a promised surprise gift for good PM performance. Based on the literature, main effects of age in PM were expected, as 7-year-olds should outperform the 5-year-olds [5, 7, 20]. Furthermore, according to the ‘reflexive-associative’ hypothesis [24] high cue-intention association was supposed to result in enhanced PM performance, for both groups. It was further expected that the promise of a ‘surprise’ reward would lead to high motivation, and therefore result in better PM performance, compared to those in the no reward condition [32]. In light of previous research, demonstrating that younger children benefit more from task conditions that reduce executive demand and/or encourage allocation of executive resources to a PM task [9], both age x reward and age x cue-intention association interactions were predicted; specifically, that the younger children were expected to benefit more than the older children from both a promised reward and cues associated with the intention.

## Method

### Participants

Seventy-nine children were recruited from local German kindergarten and primary schools to participate in the study. Two age groups were formed: a younger age group consisting of thirty-nine 5-year-olds (22 boys; M_age_ = 5.06, SD = .03) and an older age group consisting of forty 7-year-olds (14 boys; M_age_ = 7.06, SD = .03); gender distribution did not differ significantly between groups, χ^2^(1) = 3.65, *p* > .05. All children were native German speakers, were in good health and had no psychiatric, neurological or development disorders. Groups were parallel for verbal ability, measured by means of age-appropriate assessments, i.e., the verbal subtest from the German version of the Wechsler preschool and primary scale of intelligence (HAWIVA-III; [42]) for 5-year-olds (*M* = 11.18, *SD* = 2.34) and the German version of the Wechsler Intelligence Scale for Children (HAWIK-IV; [43]) for 7-year-olds (*M* = 11.43, *SD* = 2.28), with no significant differences on normed scores emerging between groups, *F*< 1. The study was approved by ethics committee of the medical faculty of the Technische Universitaet Dresden (the Ethikkommission der Medizinischen Fakultät), and children only participated after parents had provided written consent.

A 2 X 2 X 2 mixed factorial design was employed, in which cue-association (non-associated vs. associated) was varied within-subjects, and age (5-year-olds vs. 7-year-olds) and motivation (no-reward vs. reward) were varied between-subjects. Children were all tested individually.

## Materials and Procedure

The procedure of the session was similar to that of Kvavilashvili, Messer [7] in that children were asked to name picture cards (derived from the Snodgrass and Vanderwart [44] picture set) for a hand puppet named Morris the Mole but asked to respond differently to given target pictures. Children were first introduced to the experimenter and asked if they would like to stay and play some games. Upon confirmation, children were then engaged in a short conversation to ensure they felt at ease. They were then introduced to ‘Morris the Mole’ and informed that he was only young and so was excited to learn about the world. To do this he would very much like to learn what the pictures were on the cards but he needed the children to name them for him as he had poor eyesight (OT). All children were happy to help Morris and, after practicing with 2 cards, they proceeded to name the remaining 10 cards of the single task block, providing a measure of baseline OT ability.

Children were then given the PM instruction, and were introduced first to either the low cue-intention association condition (Lo-Assn), or the high cue-intention association condition (Hi-Assn) and were told that they would name some more cards for Morris again soon (30 cards for 5-year-olds and 40 cards for 7-year-olds). Piloting had shown that increasing the number of cards for 7-year-olds would ensure a similar testing duration; it had also shown that children of both age groups were able to name the majority of different pictures. The children were then told, however, that next time the card naming would be more difficult: if they were to see a picture of a certain category (an animal in the Lo-Assn block; a fruit in the Hi-Assn block) they were to name the card as usual, but then to also say ‘juice’. There were a total of 6 PM target pictures per condition (fruits: apple, banana, lemon, orange, pear and grapes; animals: dog, cat, giraffe, horse, rabbit and elephant). To ensure that all children knew the target pictures were indeed part of the relevant category, before instructing the PM task a short conversation ensued whereby children were asked to name different fruits or animals. If not all of the target pictures had been named, the experimenter prompted with questions, such as “*how about a strawberry*, *is that a fruit*?*”* Once the children had shown they knew the target pictures to be part of the relevant category, a practice block was conducted, whereby children named three cards, with the third being a target item. All children demonstrated understanding by successfully first naming the card, and then saying ‘juice’ on presentation of the target item.

Around half of the children (reward condition: 19 5-year-olds, 19 7-year-olds) were then told that, if they did very well in remembering to say ‘juice’ at the right moment then, before they went back to their friends, they would get to choose a prize from the exciting ‘surprise box’ in which there were many exciting items. To introduce a delay between PM task instruction and execution [45] the children were told they would first play another game in which they would explain the meaning of different words to Morris (HAWIVA-III and HAWIK-IV). After approximately three minutes the children were told they had done very well but it was now time to play the game they discussed earlier, and the experimental dual-task block commenced (the children were not reminded of the PM task at this point). This procedure was then repeated for the second block. Presentation of the Lo-Assn and Hi-Assn task blocks was counter-balanced. Once both blocks were completed, any remaining ability tasks were finished, after which all children were congratulated on an excellent performance and told that Morris was very grateful for their help, and that they could choose something from the surprise box (irrespective of reward condition).

Children’s PM performance was measured as the number of correct responses (max. 6 for each association condition) that they remembered to say ‘juice’ after presentation of the correct picture cue. Dependent variables for OT performance were percentages of correctly named picture cards (for the baseline and dual taskblocks, respectively).

## Results

### PM performance

Table 1 shows the mean percentage of successful PM hits by age, reward and cue-intention association. A 2 (age: 5- vs. 7-year-olds) by 2 (reward: no reward vs. reward) by 2 (cue-intention association: Lo-Assn vs. Hi-Assn) mixed analysis of variance (ANOVA) with repeated measures on the last factor revealed a significant main effect of age. The 7-year-olds (*M* = 74.17, *SD* = 37.02) performed better than the 5-year-olds (*M* = 48.29, *SD* = 38.67) on the PM task, *F*(1, 75) = 9.70, *p* < .01 $\eta_{p}^{2}=.11$. Further, there was a main effect of reward, with those in the reward condition (*M* = 71.27, *SD* = 34.37) performing better than those in the no reward condition (*M* = 52.24 *SD* = 42.62) on the PM task *F*(1, 75) = 5.46, *p* = .02, $\eta_{p}^{2}=.07$. However, the level of cue-intention association did not affect performance, (*F* < 1) and there were no significant interactions between any of the three factors (all *Fs* < 1.02).

**Table 1:** Mean percentage (and standard deviation) of successful PM hits by task block and condition.

	No Reward		Reward	
	Lo-Assn (non-associated)	Hi-Assn (associated)	Lo-Assn (non-associated)	Hi-Assn (associated)
5-year-olds	39.17 (41.63)	32.50 (43.08)	59.65 (46.25)	63.16 (44.99)
7-year-olds	63.49 (47.03)	72.22 (42.27)	78.07 (36.45)	84.21 (30.16)
Total	51.63 (45.61)	52.85 (46.68)	68.86 (42.12)	73.68 (39.26)

As gender distribution was not perfectly balanced between groups (despite not being statistically different) gender was added as an additional factor in a 2 (Age: 5- vs. 7-year-olds) by 2 (Reward: No reward vs. Reward) by 2 (Gender: Boys vs. Girls) by 2 (Cue-intention association: Lo-Assn vs. Hi-Assn) mixed analysis of variance (ANOVA), to ensure gender did not influence the between-group PM performance differences. The results revealed persisting main effect of age (*p* < .01) and Reward (*p* < .05), but no main effect of Gender (*p* = .835). Further, no interactions between Gender x Group or Gender x Reward were found (all *F*s < 1), implying that the between-group differences were not an artefact of gender.

### OT performance

Table 2 shows the mean percentage of successful OT hits by age, reward and cue-intention association. A 2 (age: 5- vs. 7-year-olds) by 2 (reward: no reward vs. reward) by 3 (block: baseline vs. Lo-Assn vs. Hi-Assn) ANOVA, with repeated measures on the last factor, revealed significant main effects of age *F*(1, 75) = 134.699, *p* < .001, $\eta_{p}^{2}=.642$, block *F*(1.65, 124.01) = 615.960, *p* < .001, $\eta_{p}^{2}=.89$ and reward *F*(1, 75) = 8.555, *p* < .01, $\eta_{p}^{2}=.102$. No significant interaction was found between all three factors, but a significant interaction was found between age and block, *F*(1.65, 124.01) = 17.458, *p* < .001, $\eta_{p}^{2}=.189$; pairwise comparisons showed that both 5yr- and 7yr-olds achieved a higher percentage of correctly named cards in baseline block than both other blocks (all *ps* < .001) but only 5yr-olds performed better in the Hi-Assn block than the Lo-Assn block (*p* < .05). A further interaction was found between age and reward, *F*(1, 75) = 6.55, *p* < .05, $\eta_{p}^{2}=.08$. Pairwise comparisons revealed 5yr-olds, but not 7yr-olds (*p*>.05), achieved a higher percentage of correctly named cards in the reward condition, compared to the no reward condition (*p* < .001). Finally, a significant interaction was found between block and reward, *F*(1.65, 124.01) = 3.696, *p* < .05, $\eta_{p}^{2}=.047$. Pairwise comparisons revealed that, whilst both the reward group and the no reward group performed better in baseline performance than in both subsequent assn-trial blocks (all *ps* < .05) those in the reward condition named a higher percentage of cards in the baseline block than those in the no reward condition (*p* < .01; mean difference = 3.34%). However, both groups performed equally well in the subsequent Assn-trial blocks (all *ps*>.05). To ensure that card-naming ability did not confound the effects found in the PM performance, we included the baseline performance as a covariate in the PM mixed ANOVA; both the PM Group effect (*p* = .002) and the PM Reward effect (*p* = .016) remained significant. We also conducted an independent samples *t*-test to ensure both reward groups were parallel for standardized verbal ability. Results revealed no significant differences between the groups, *t*(77) = -.631, *p* = .530

**Table 2:** Mean percentage (and standard deviation) of successful OT naming trials by task block and condition of reward.

	No Reward			Reward		
	Baseline	Lo-Assn (non-associated)	Hi-Assn (associated)	Baseline	Lo-Assn (non-associated)	Hi-Assn (associated)
5-year-olds	93.50 (6.71)	73.17 (5.24)	75.33 (3.65)	98.42 (5.02)	76.14 (3.73)	76.67 (3.14)
7-year-olds	97.62 (5.39)	83.93 (1.69)	84.29 (1.16)	99.47 (2.29)	83.42 (1.90)	83.55 (1.92)
Total	95.61 (6.34)	78.68 (6.64)	79.92 (5.25)	98.95 (3.88)	79.78 (4.70)	80.11 (4.33)

## Discussion

The current study is the first to examine the effects of age and cue-intention association on PM in 5- and 7-year-old children, whilst being only the second to investigate the effects of providing an extrinsic incentive in this age group. Based on the literature, primarily the Executive Framework [12] and Multiprocess Framework [17], it was expected that 7-year-old children would outperform 5-year-old children in the PM task, across both conditions. Further, it was anticipated that both the promise of reward, in the form of a gift incentive, and high cue-intention association would positively affect PM performance, and that these effects would possibly more benefit the younger children, as reward would encourage the allocation of EF resources to the task, and high cue-intention association would promote reflexive-associative (i.e. more automatic) processes, reducing EF demand.

Firstly, as expected, significant age effects were found, with the 7-year-olds outperforming 5-year-olds in the PM task, across both conditions. Also in line with predictions and previous evidence of positive effects of motivation on PM in children [29, 31, 32] those who were promised a reward performed better than those who were not, an effect found across age groups. This result is in contrast to the only other study to specifically investigate the effects of extrinsic incentives on PM in young children [23]. It seems possible that by reducing the working memory demands of the OT in the current study, compared to those of Guajardo and Best’s study [23] (naming, rather than remembering, cards) potentially confounding EF factors were reduced; and by providing a more motivating incentive (a ‘surprise box’ rather than common food and toy items) increased motivation was achieved. It is therefore argued that the current results extend literature on the positive effects of motivation on PM found in tasks that were more intrinsically motivating [29, 31, 32], demonstrating that extrinsic rewards may also be beneficial to PM performance. At first glance, these results may indicate that children focused more on the PM task when offered a reward. However, the apparent lack of additional OT costs in the reward condition does not support this notion. In fact, for 5-year-olds, OT performance was actually better in the reward condition. This is perhaps surprising as the Multiprocess Framework [17] posits that perceived task importance influences the allocation of attentional resources between the OT and PM task, thus benefitting the ‘more important’ task at a cost to the other task, an effect which has been shown in previous studies [46–49]. Therefore, if the reward increased motivation due to the perceived importance of the PM task, one might have expected to see an increased cost to the OT. However, we contend that, even if attention was drawn to the more important PM task, it is unlikely that the deliberately simple card-naming nature of the OT would be adversely affected. Indeed, most OT errors were simple misnaming errors, for example, calling an orange an apple. It is possible, however, that the children were slower to name the cards, and so future studies should employ more sensitive measures (e.g., reaction times) which may reveal an OT cost. This is an important future direction, which could elucidate upon the effect of additional PM tasks and their importance relative to the OT, and the impact they may have on the different phases of the PM process (e.g. [12]). For example, other recent studies which also did not find a cost to OT, despite a positive ‘importance’ effect on the PM task in Parkinson’s patients, or positive effects of monetary incentives in healthy populations ([34, 50] respectively) posited it is possible that increased personal importance of the intention resulted in a stronger encoding of the PM cues at the time of instruction, thus facilitating PM cue retrieval without requiring further attentional resources, a notion further supported by the motivational-cognitive model of Penningroth and Scott [33]. It would be interesting to investigate this further by increasing the demand/difficulty of the OT, and observe how this interacts with rewards.

This finding has important implications for the home and school environment of young children, where incentives (and motivation) could be employed as a strategy for remembering important everyday prospective tasks, such as remembering to give a letter to a parent, or bringing a swimming kit in on the appropriate day. This could also apply to remembering to execute appropriate social behaviours at the right time, such as saying ‘please’ and ‘thank-you’, or putting a hand up before giving an answer; in fact, encouraging good social behaviour through proactive strategies (e.g., increasing motivation through rewards such as praise and stickers) have been argued to be the most common, and effective, good-behaviour strategies in schools [51, 52]. However, with such paucity of PM research in this area, it is important for future research to further investigate motivation and incentives. Indeed, this may be particularly important for populations with social impairments, such as those with autism spectrum disorder [53] who may be less socially motivated to successfully perform a PM task (e.g. to remember to perform an action in the future in such a way as to consider the well-being of others, such as closing a door quietly if a classmate has a headache) and thus benefit more from an incentive conferring motivation on a personal level.

Perhaps the most surprising result was found in the cue-intention association condition, where participants of both ages did not, as predicted, perform significantly better in the high-association condition, compared to the low-association condition. This prediction was derived from the previous positive cue-intention association effects found in both healthy adults [24, 37, 38] and clinical groups [39] which suggested that high-association enables more reflexive/automatic processes [17], reduces EF demands and thus improves the PM performance, particularly for those with less-developed EF resources, e.g., younger children [20]. However, the present finding of no effects of association on PM performance is in line with other previous evidence [54]. Another possibility is that the current result could be explained by the ‘delay-execute’ effect [55, 56]; previous research has found that requiring participants to delay executing an intention once it is retrieved negatively affects PM performance [55, 56]. This effect is theorized to arise due to increased demands on working memory, task switching and inhibition when prospective intentions cannot be performed immediately upon appearance of the target cue [57–60]. Children in the current study were instructed, on presentation of the target item, to first continue with the OT of naming the card, and *then* to execute the PM task of saying ‘juice’. However, it is possible that including this in the design of the study inadvertently introduced a delay-execute effect, which neutralized the positive effect of high cue-intention association. In other words, increasing the cue-intention association in the high association condition may have induced a stronger reflexive retrieval response, *but requiring its initial inhibition* upon cue presentation may have placed a proportionately higher demand on inhibition and working memory as compared to the low association condition. More specifically, the strong reflexive response of saying juice when seeing the strongly associated cue of a fruit, may have been neutralized by the ‘delay-execute’ effect of inhibiting the intention and holding it in mind whilst first naming the card. This notion has important everyday implications, as young children arguably must often wait to execute an intention once it has been retrieved e.g., waiting for adult to finish talking/teaching before passing on a message [56]. Inhibiting an intention in this way could, for example, put a strain on working memory and induce a fear of failing the intention, which could, in turn cause anxiety, as well as significant cost to the ongoing-activity, such as sitting in lesson. This example should be considered cautiously as it is highly speculative at this point but, given the potentially important implications, future research should further examine the delay-execute effect in young children, and the ways in which it may interact with factors thought to facilitate automatic retrieval.

Finally, both age groups saw a decrease in OT performance during both the dual-task blocks (Lo-Assn and Hi-Assn) compared to the Baseline-block; furthermore, the costs to the OT were greater in the Lo-Assn block, but only for the younger children. These results are thus in line with Mahy’s Executive Framework [12] in that developing attentional resources/EF are important underlying mechanisms for the PM process in children, with those with less resources more adversely affected in their ongoing activity by the addition of an extra task. These results are also consistent with Scullin, McDaniel [18] which saw greater OT costs when strategic processes were needed to monitor for the PM cue, and less cost when automatic processes were sufficient. These data contribute further evidence for the development of PM [7–9] and are in line with both the Executive Framework [12] and the Multiprocess Framework [17] in that developing attentional resources/EF are important underlying mechanisms for PM in children, with those with less resources more adversely affected by the addition of an extra task.

In conclusion, the current study is the first to provide evidence for the benefits of incentives on PM in 5- and 7-year-old children, whilst also contributing further evidence in support of the important role that executive functioning plays in the PM process, as posited by the Executive Framework and the Multiprocess Framework [17]. Furthermore, the results regarding the lack of cue-intention association introduced the possibility that ‘delay-execute’ effects can neutralize the benefits of automatic processes, which would have important implications for children’s everyday PM functioning, opening up important future directions for research.

## Supporting Information

S1 Dataset This includes the data collected and analysed for the current study. (SAV)
